# Comparing laminectomy and unilateral hemilaminectomy in spinal hemangioblastoma resection: A multicenter study

**DOI:** 10.1016/j.bas.2026.106004

**Published:** 2026-03-05

**Authors:** Johannes Wach, Alim Emre Basaran, Martin Vychopen, Tarik Tihan, Maria Wostrack, Vicki M. Butenschoen, Bernhard Meyer, Sebastian Siller, Nils Ole Schmidt, Julia Onken, Peter Vajkoczy, Alejandro N. Santos, Laurèl Rauschenbach, Philipp Dammann, Ulrich Sure, Jan-Helge Klingler, Roberto Doria-Medina, Jürgen Beck, Bianca-Ioana Blaß, Christine Juliane Gizaw, Romina Hohenhaus, Sandro M. Krieg, Obada T. Alhalabi, Lukas Klein, Claudius Thomé, Nikolaus Kögl, Przemyslaw Kunert, Tomasz Czernicki, Tobias Pantel, Maximilian Middelkamp, Sven Oliver Eicker, Ahed H. Kattaa, David J. Park, Steven D. Chang, Fatma Kilinc, Marcus Czabanka, Erdem Güresir

**Affiliations:** a Department of Neurosurgery, University Hospital Leipzig, Leipzig, Germany; b Comprehensive Cancer Center Central Germany, Partner Site Leipzig, Leipzig, Germany; c Division of Neuropathology, UCSF School of Medicine, San Francisco, CA, USA; d Department of Pathology, Miller School of Medicine, University of Miami, Florida, USA; e Department of Neurosurgery, Technical University of Munich, School of Medicine, Klinikum rechts der Isar, Germany; f Department of Neurosurgery, University Hospital, University of Regensburg, Regensburg, Germany; g Department of Neurosurgery, Charité - Universitätsmedizin Berlin, Corporate Member of Freie Universität Berlin and Humboldt-Universität zu Berlin, Berlin, Germany; h Department of Neurosurgery and Spine Surgery, University Hospital Essen, Germany; i Department of Neurosurgery, Royal Adelaide Hospital, Australia; j DKFZ-Division Translational Neurooncology at the WTZ, DKTK Partner Site, University Hospital Essen, Germany; k Department of Neurosurgery, Medical Center – University of Freiburg, Germany; l Department of Neurosurgery, Heidelberg University Hospital, Heidelberg, Germany; m Medical Faculty, Heidelberg University, Heidelberg, 69120, Germany; n Department of Neurosurgery, Medical University Innsbruck, Innsbruck, Austria; o Department of Neurosurgery, Medical University of Warsaw, Poland; p Department of Neurosurgery, University Medical Center Hamburg-Eppendorf, Hamburg, Germany; q Department of Spine and Scoliosis Surgery, Lubinus Clinicum, 24106 Kiel, Germany; r Department of Neurosurgery, Stanford University School of Medicine, Stanford, CA, USA; s Department of Neurosurgery, University Hospital Frankfurt, Frankfurt am Main, Germany; t Department of Neurological Surgery, University of Miami Miller School of Medicine, Miami, USA; u Department of Neurosurgery, Klinikum Main-Spessart, Lohr am Main, Germany

**Keywords:** Hemilaminectomy, Laminectomy, Multicenter study, Spinal hemangioblastoma, Surgical outcomes

## Abstract

**Purpose:**

This study compared laminectomy and hemilaminectomy for resection of spinal (sHBs), evaluating extent of resection, 12-month postoperative functional outcomes, perioperative complications overall, with particular attention to postoperative bleeding.

**Material and methods:**

This retrospective international multicenter study included 280 primary sHB patients from 13 neuro-oncological centers who underwent either laminectomy (n = 125) or hemilaminectomy (n = 155). The endpoints were the extent of resection, functional outcomes at 12 months, and postoperative bleeding requiring retreatment. Multivariable logistic regression analysis was performed to determine independent risk factors associated with these outcomes.

**Results:**

The rate of complete resection was similar between both surgical approaches, with 86.4% in the laminectomy group and 90.3% in the hemilaminectomy group (*p* = 0.35). Independent predictors of incomplete resection included preoperative modified McCormick >2 (OR: 4.29, *p* = 0.001), combined intra- and extramedullary tumor location (OR: 2.91, *p* = 0.03), and cervical or thoracic tumor location (OR: 3.38, *p* = 0.01). Functional outcomes at 12 months did not differ significantly between the laminectomy- and hemilaminectomy-groups (*p* = 0.97). Postoperative bleeding was more frequently observed in tumors involving two or more spinal segments (OR: 14.6, *p* = 0.01). The choice of surgical approach did not impact the incidence of postoperative bleeding (*p* = 0.55).

**Conclusion:**

Laminectomy and hemilaminectomy result in comparable outcomes of sHB. Tumors affecting multiple spinal segments are associated with an increased risk of postoperative bleeding, while combined intra- and extramedullary growth, impaired preoperative functioning and non-lumbar location were associated with incomplete resection. Given the comparable outcomes, the selection of the surgical approach may be guided by surgeon preference and individual patient anatomy.

## Highlights

• Laminectomy and hemilaminectomy achieve comparable rates of complete resection in spinal hemangioblastomas.
• Multisegmental tumor involvement is the main predictor of postoperative bleeding, independent of approach.
• Incomplete resection is primarily associated with tumor location and preoperative neurological impairment rather than surgical technique.

## Graphical abstract

**Figure:**
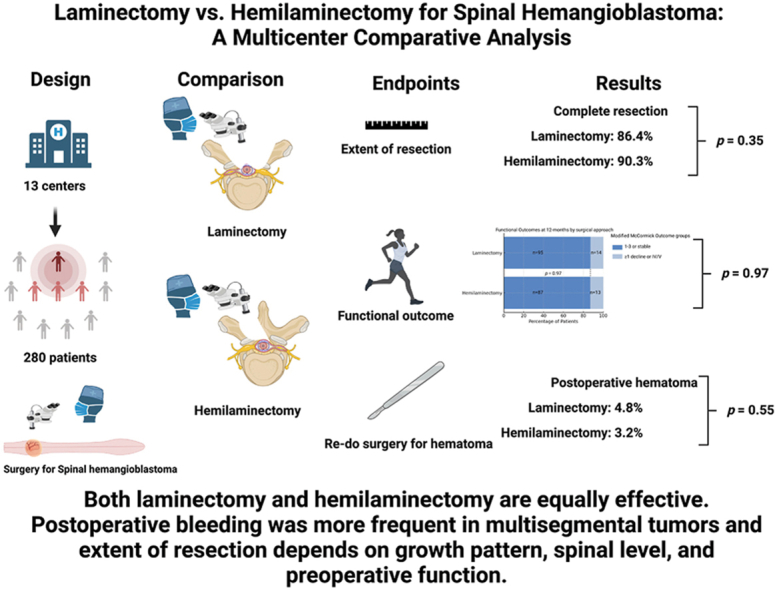

## 1 Introduction

Spinal hemangioblastomas (sHBs) are rare, highly vascular tumors that pose significant surgical challenges due to their location and potential for neurological impairment (Deng et al., 2014; Wach et al., 2025a). Traditionally, laminectomy has been the standard approach for resecting these lesions, providing broad exposure but often at the cost of spinal instability, kyphosis and increased postoperative pain, particularly in cervical location (Chiou et al., 1989; Furtado et al., 2011). In contrast, hemilaminectomy, a less invasive alternative, regularly preserves the spinous process and both supra- and interspinous ligaments and may reduce the risk of kyphosis, pain, and instability (Millward et al., 2015).

Our previous multicenter study on sHBs has highlighted the importance of complete resection in optimizing long-term tumor control in both sporadic and von Hippel-Lindau (vHL)-associated sHBs (Wach et al., 2025a). However, the impact of surgical approach on extent of resection, postoperative hematoma requiring retreatment and functional outcome remains unclear for this disease, as well as for spinal intradural tumors in general, so far. The present evidence comparing hemilaminectomy and laminectomy for spinal intradural tumor surgery is limited to smaller monocentric cohorts and heterogeneous collectives with various tumor entities such as spinal schwannomas and meningiomas (Onishi et al., 2025; Muncan et al., 2024).

The present investigation represents a prespecified secondary analysis from a multicentric study comparing laminectomy and hemilaminectomy in a homogeneous cohort of primary surgery for sHB. (Wach et al., 2025a). The primary aim of this study was to compare extent of resection between laminectomy and hemilaminectomy in primary sHB surgery. Secondary aims were to compare postoperative bleeding requiring surgical retreatment and functional outcome at 12 months.

## 2 Methods

Clinical data were gathered following approval from the respective institutional review boards and subsequently centralized for analysis at Leipzig University. The study was conducted in accordance with ethical standards and received approval from the Leipzig University Ethics Committee (No.: 382/23-ek). Adherence to the STROBE guidelines for observational cohort studies was ensured (see Supplementary Table 1). Further details regarding the study protocol, patient population, imaging criteria, defined endpoints, and statistical methodology were previously described (Wach et al., 2025a).

### 2.1 Study population

The pooled database of thirteen neuro-oncological centers in the United States and in Europe were searched for primary sHB patients between January 1997 and August 2023. Patients who underwent either only hemilaminectomy or laminectomy without additional procedures such as laminoplasty or fixation. The primary study cohort included the comparison between laminectomy and hemilaminectomy. The included patients were consecutively managed at the individual centers. The approach (laminectomy vs. hemilaminectomy) was selected by the treating neurosurgeon according to local standards and case-specific considerations (e.g., tumor location, segmental extension, lateralization, and anticipated need for exposure). Patients who underwent surgery for a recurrent sHB were excluded. As previously outlined, standardized demographic, clinical, pathological, and imaging data were retrieved from the databases for analysis (Wach et al., 2025a). Patients who underwent laminoplasty were excluded from the primary two-group comparison (laminectomy vs. hemilaminectomy) to preserve methodological homogeneity; however, laminoplasty cases were analyzed separately in a predefined subgroup analysis.

### 2.2 Surgical procedures/approaches

The laminectomy procedure was defined as a removal of the spinous process and a bilateral removal of the vertebral laminae, whereas the hemilaminectomy procedure was defined as the unilateral removal of the vertebral laminae centered above the intraspinal tumor. Flavectomy was then performed to expose the spinal canal. Additionally, the spinous process was partially thinned using a high-speed drill for undercutting. In case of more lateral tumor location, the exposure may include partial facetectomy. In laminoplasty, the cut laminae and spinous processes are repositioned and secured using screws and connectors, while the supraspinal ligament is stabilized with sutures.

## 3 Endpoints

Primary endpoint**:** extent of resection (complete vs. incomplete).

Secondary endpoints**:** (i) postoperative bleeding requiring retreatment and (ii) functional outcome at 12 months assessed by modified McCormick Scale.Contrast-enhanced MRI scans were assessed by radiologists and neurosurgeons at each participating center. Complete resection (CR) was defined as the total removal of the tumor with no detectable residual tissue on postoperative imaging (Wach et al., 2025a). Functional status was assessed using the modified McCormick Scale (mMCS). The mMCS categorizes mobility as follows: Grade I – normal gait, Grade II – mild gait impairment without the need for support, Grade III – gait requiring support, Grade IV – mobility dependent on assistance, and Grade V – wheelchair dependence (McCormick et al., 1990). Functional outcome was evaluated at the 12-month follow-up using predefined outcome groups: the Good (G) group, which included patients with McCormick Scale grades I–III or stable grades**,** and the Poor (P) group, defined as those with a decline of at least one grade or remaining in grades IV/V (Tsuji et al., 2020).

### 3.1 Statistical analysis

Univariable statistical analyses were performed using Pearson's chi-squared test or Fisher's exact test for nominal variables and Student's t-test for metric variables, with all *p*-values reported as two-sided. Receiver-operating characteristic (ROC) analysis was performed to identify optimum cut-off value of metric variables for binary analysis. Multivariable binary logistic regression analyses were conducted to identify factors associated with extent of resection and postoperative bleeding. All variables with a significant *p*-value (<0.05) in the univariable analysis were included in the multivariable analysis. Additionally, given the primary hypothesis and the comparative nature of the study, the surgical approach (laminectomy vs. hemilaminectomy) was incorporated into the model regardless of its significance in univariable analysis. Sankey plots were generated using RAWGraphs and further refined with BioRender.^10^ Staked bar charts and forests plots were created using Python's Matplotlib package.

## 4 Results

### 4.1 Patient selection

**Fig. 1:**
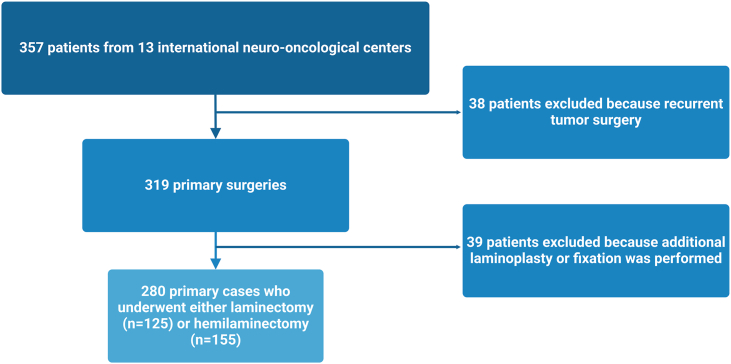
Flowchart depicting the patient selection process.

A total of 357 patients from 13 international neuro-oncological centers were initially considered for inclusion in this study. After excluding 38 patients due to prior surgery for tumor recurrence and 39 patients who underwent additional laminoplasty or spinal fixation, 280 patients remained eligible for analysis. Among them, 125 patients (44.6%) underwent laminectomy, while 155 patients (55.4%) underwent hemilaminectomy (Fig. 1).

### 4.2 Patient characteristics among the laminectomy or hemilaminectomy groups

The mean age of the entire cohort was 43.1 ± 16.3 years, with no significant difference between groups (*p* = 0.48)**.** Female patients accounted for 41.6% in the laminectomy group and 51.0% in the hemilaminectomy group (*p* = 0.12). Preoperatively, patients with McCormick grades I-II comprised 63.7% of the laminectomy group and 73.0% of the hemilaminectomy group (*p* = 0.12).

The distribution of tumor location varied between groups. Thoracic tumors were more frequent in the laminectomy group (34.4%) compared to the hemilaminectomy group **(**23.2%, *p* = 0.053**)**, while tumors at the thoracolumbar junction were more common in the hemilaminectomy group (13.5% vs. 3.2%, *p* = 0.003)**.** Laminectomy was performed across more levels, with 32.8% involving two levels and 15.2% involving three levels, compared to 20.6% and 11.6% in the hemilaminectomy group, respectively (*p* = 0.03 and *p* = 0.48)**.** The extent of resection, tumor characteristics, and operative factors are detailed in Table 1.

**Table 1:** Patient characteristics.

Variable	Laminectomy (n = 125)	Hemilaminectomy (n = 155)	p-value
Age, mean years (SD)	43.9± 17.2	42.5±15.7	0.48
Female, n patients (%)	52 (41.6%)	79 (51.0%)	0.12
Modified McCormick, preoperative, n patients (%),^a^			0.12
I-II	79 (63.7%)	111 (73.0%)	
III-V	45 (36.3%)	41 (27.0%)	
Location			
Cervical	57 (45.6%)	74 (47.4%)	0.39
Cervicothoracic	12 (9.6%)	12 (7.7%)	0.67
Thoracic	43 (34.4%)	36 (23.2%)	0.053
Thoracolumbar	4 (3.2%)	21 (13.5%)	0.003
Lumbar	7 (5.6%)	8 (5.2%)	0.99
Lumbosacral	2 (1.6%)	4 (2.6%)	0.70
Levels operated, n (%)			
1	56 (44.8%)	104 (67.1%)	0.003
2	41 (32.8%)	32 (20.6%)	0.03
3	19 (15.2%)	18 (11.6%)	0.48
4	9 (7.2%)	1 (0.6%)	0.002
Location			
Intramedullary	88 (70.4%)	113 (72.9%)	0.74
Extramedullary	19 (15.2%)	19 (12.3%)	0.53
Combined	17 (13.6%)	23 (14.8%)	0.98
Extent of resection			
Complete	108 (86.4%)	140 (90.3%)	0.35
Incomplete	17 (13.6%)	15 (9.7%)	

a available in 276 patients.

### 4.3 Extent of resection and predictors of incomplete resection

Among 280 patients, 32 (11.4%) had incomplete resection, while 248 (88.6%) achieved complete resection. Patients with incomplete resection were older (48.9 ± 16.2 vs. 42.4 ± 16.3 years, *p* = 0.03) and more often had a preoperative McCormick grade >2 (59.4% vs. 28.6%, *p* = 0.001). Thoracic tumors were more frequent in the incomplete resection group (53.1% vs. 25.0%, *p* = 0.0003), while cervical tumors were less common (15.6% vs. 50.8%, *p* = 0.0001). Combined intra- and extramedullary tumors were associated with incomplete resection (31.3% vs. 12.1%, *p* = 0.01). The number of operated levels and surgical approach were not significantly different between groups. Table 2 summarizes the univariable analysis.

**Table 2:** Patient-, disease-, and treatment-specific characteristics among incompletely or completely resected spinal hemangioblastoma patients.

Variable	Incomplete resection (n = 32)	Complete resection (n = 248)	p-value
Age, mean years (SD)	48.9±16.2	42.4±16.3	0.03
Female, n patients (%)	14 (43.8%)	18 (56.3%)	0.72
Location			
Cervical	5 (15.6%)	126 (50.8%)	0.0001
Cervicothoracic	0 (0.0%)	24 (9.7%)	0.09
Thoracic	17 (53.1%)	62 (25.0%)	0.0003
Thoracolumbar	4 (12.5%)	21 (8.5%)	0.50
Lumbar	4 (12.5%)	11 (4.4%)	0.08
Lumbosacral	2 (6.3%)	4 (1.6%)	0.14
Levels operated, n (%)			
1	14 (43.8%)	146 (58.9%)	0.13
2	10 (31.3%)	63 (25.4%)	0.52
3	6 (18.8%)	31 (12.5%)	0.40
4	2 (6.3%)	8 (3.2%)	0.14
Location			
Intramedullary Extramedullary Combined	18 (56.3%)	183 (74.1%)	0.06
	4 (12.5%)	34 (13.8%)	0.99
	10 (31.3%)	30 (12.1%)	0.01
Surgical approach			
Laminectomy	17 (53.1%)	108 (43.5%)	0.35
hemilaminectomy	15 (46.9%)	140 (56.5%)	

**Fig. 2:**
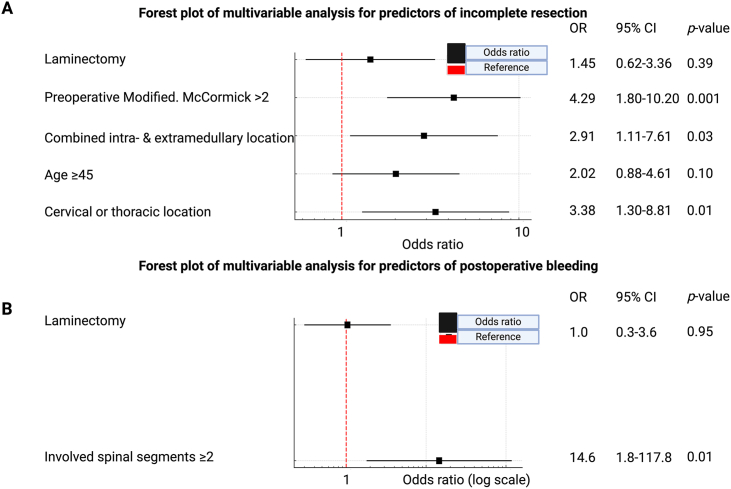
Forest plots illustrating predictors of incomplete resection (**A**) and postoperative bleeding (**B**). Each box represents the odds ratio, with whiskers indicating the 95% confidence interval. The red dashed line marks the reference value (OR = 1). Significant predictors of incomplete resection included preoperative functional impairment, combined intra- and extramedullary tumor location, and cervical or thoracic tumor location. Postoperative bleeding was significantly associated with multisegmental tumor involvement, while the surgical approach showed no significant effect on either outcome.

Complete resection was achieved in 86.4% of patients undergoing laminectomy and 90.3% of those undergoing hemilaminectomy **(***p* = 0.35**)**. Significant variables from univariable analysis and surgical approach were considered in the multivariable binary logistic regression analysis. Age was dichotomized into </≥45 years at diagnosis based on the ROC curve analysis (see Supplementary Fig. 1). Multivariable analysis identified preoperative mMCS >2 (OR: 4.29, 95% CI: 1.80-10.20, *p* = 0.001)**,** combined intra- and extramedullary tumor growth (OR: 2.91, 95% CI: 1.11-7.61, *p* = 0.03)**,** and tumors located in the cervical or thoracic spine (OR: 3.38, 95% CI: 1.30-8.81, *p* = 0.01) as significant predictors of incomplete resection. The choice of laminectomy or hemilaminectomy did not significantly impact resection completeness (*p* = 0.39, Fig. 2A).

### 4.4 Postoperative bleeding and risk factors

Among 280 patients, 11 (3.9%) experienced postoperative bleeding. Patients with bleeding had a lower mean age (39.5 ± 15.8 vs. 43.3 ± 16.4 years, *p* = 0.44) and a lower proportion of females (27.3% vs. 47.6%, *p* = 0.23), though these differences were not statistically significant. Thoracic tumors were more common in the bleeding group (54.5% vs. 27.1%, *p* = 0.08). Tumors involving two or more spinal segments were significantly associated with postoperative bleeding (72.7% vs. 24.2%, *p* = 0.001), while single-segment tumors were more frequent in patients without bleeding (59.1% vs. 9.1%, *p* = 0.0007). No significant association in univariable analysis was found between bleeding and tumor location or surgical approach (see Table 3). While the surgical approach did not influence the likelihood of postoperative bleeding, multivariable regression analysis revealed that tumors involving ≥2 spinal segments were significantly associated with a higher risk of postoperative hemorrhage (OR: 14.6, 95% CI: 1.8-117.8, *p* = 0.01) (Fig. 2B).

**Table 3:** Patient-, disease-, and treatment-specific characteristics among those with or without postoperative bleeding.

Variable	Postoperative bleeding (n = 11)	No postoperative bleeding (n = 269)	p-value
Age, mean years (SD)	39.5±15.8	43.3±16.4	0.44
Female, n patients (%)	3 (27.3%)	128 (47.6%)	0.23
Modified McCormick, preoperative, n patients (%),^a^			0.51
I-II	6 (60.0%)	184 (69.2%)	
III-V	4 (40.0%)	82 (30.8%)	
Location			
Cervical	3 (27.3%)	128 (47.6%)	0.23
Cervicothoracic	0 (0.0%)	24 (8.9%)	0.61
Thoracic	6 (54.5%)	73 (27.1%)	0.08
Thoracolumbar	0 (0.0%)	25 (9.3%)	0.61
Lumbar	2(18.2%)	13 (4.8%)	0.11
Lumbosacral	0 (0.0%)	6 (2.2%)	0.99
Levels operated, n (%)			
1	1 (9.1%)	159 (59.1%)	0.0007
2	8 (72.7%)	65 (24.2%)	0.001
3	1 (9.1%)	36 (13.4%)	0.99
4	1 (9.1%)	9 (3.3%)	0.33
Location			
Intramedullary	7 (63.6%)	194 (72.4%)	0.51
Extramedullary	0 (0.0%)	38 (14.2%)	0.37
Combined	4 (36.4%)	36 (13.4%)	0.06
Surgical approach	6 (54.5%)	119 (44.2%)	0.55
Laminectomy	5 (45.5%)	150 (55.8%)	
hemilaminectomy			

a available in 276 patients.

### 4.5 Functional outcomes at 12-months after surgery

**Fig. 3:**
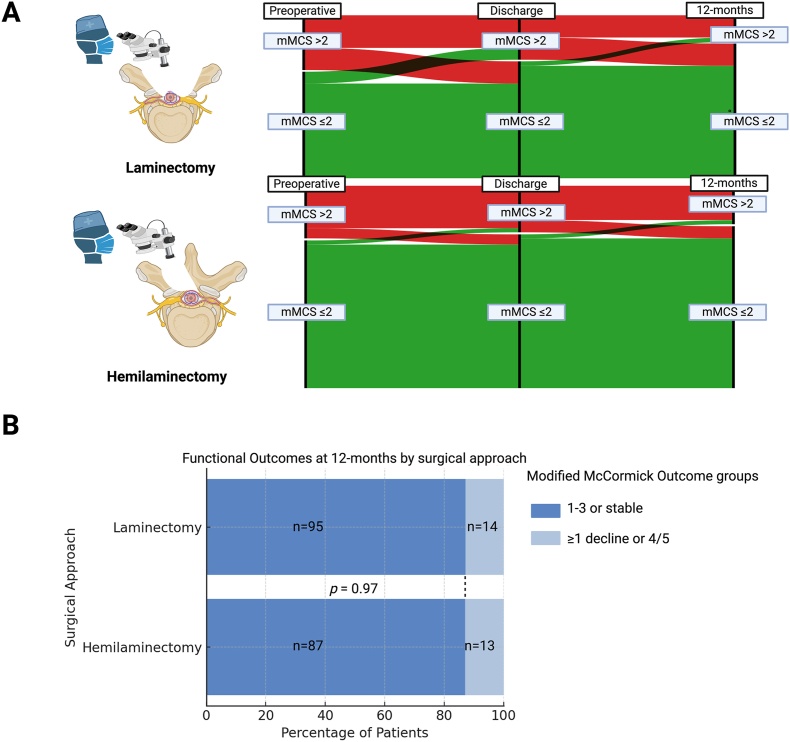
Functional outcomes at 12-month follow-up for patients undergoing laminectomy or hemilaminectomy. (**A**) Sankey plots depicting the longitudinal progression of functional status from the preoperative period to discharge and 12 months postoperatively, categorized by the mMCS (≤2/>2). Most patients maintained or improved their functional status, with similar distributions in both groups. (**B**) Bar chart comparing functional outcomes at 12 months, showing no significant difference between surgical approaches (*p* = 0.97). The majority of patients remained in or improved to mMCS I–III, while a smaller proportion experienced functional decline (grades IV/V).

Functional outcome was assessed using mMCS and the categorization into P- and G-group at 12-months after surgery. Preoperatively, at discharge and after 12-months the proportions of patients with a mMCS >2/≤2 were comparable among laminectomy or hemilaminectomy (see Fig. 3A). The Sankey plots show the longitudinal progression of functional status from preoperative assessment to discharge and 12-month follow-up, categorized by the mMCS. In both groups, most patients either maintained or improved their functional status over time, with no significant differences in outcome trajectories.

At discharge**,** the proportion of patients with mMCS grades I–II was slightly higher in the hemilaminectomy group (73.3%) compared to the laminectomy group (68.8%)**,** while those with mMCS >2 was slightly more frequent in the laminectomy group (31.2% vs. 26.7%). However, at 12-month follow-up, these differences had balanced out, with no significant difference between surgical approaches (p = 0.97, see Fig. 3B). The majority of patients in both groups demonstrated good functional outcomes (mMCS I–III or stable function), accounting for 82.7% of the laminectomy group and 81.2% of the hemilaminectomy group. Only a small subset of patients in each group experienced a decline in function or remained at mMCS IV/V (17.3% and 18.8%, respectively).

### 4.6 Subgroup analysis comparing hemilaminectomy with laminoplasty

The subgroup analysis between hemilaminectomy and laminoplasty showed no significant differences in key surgical outcomes. Complete resection was achieved in 90.3% of hemilaminectomy cases and 85.3% of laminoplasty cases (*p* = 0.37). Postoperative bleeding occurred in 3.2% of hemilaminectomy cases, with no reported cases in the laminoplasty group (*p* = 0.59). Although the laminoplasty group had a higher proportion of multilevel procedures (67.6% vs. 32.9%, *p* = 0.0001) and more extramedullary tumors (38.2% vs. 12.3%, *p* = 0.0008), these factors did not translate into significant differences in resection rates or postoperative bleeding risk. Supplementary Table 2 summarizes the results.

## 5 Discussion

This international multicenter study provides a comparative analysis of laminectomy and hemilaminectomy for the resection of sHBs, evaluating extent of resection, postoperative bleeding, and functional outcomes. Our findings suggest that both surgical approaches are equally effective in achieving complete resection and preserving functional status, while multisegmental tumors were associated with a higher risk of postoperative bleeding.

### 5.1 Extent of resection

The completeness of resection is a critical determinant of long-term outcomes in sHB surgery.^2^ In our cohort, CR was achieved in 86.4% of patients undergoing laminectomy and 90.3% in the hemilaminectomy group, with no significant difference between approaches. These findings align with a previous retrospective study investigating 52 patients with various spinal intradural tumors demonstrating that hemilaminectomy provides sufficient access for complete tumor removal without compromising resection rate.^11^ However, the present findings are only semiquantitatively adjusted for the tumor size by number of spinal segments involved, growth pattern (intra- and extramedullary) and level (cervical, thoracic, lumbar). The measurement of tumor volume might further refine this analysis in a future series with high-resolution thin slice spinal MRI. Additionally, our findings also confirm the conclusions of Turel et al. (2015), who reported in non-comparative single-arm retrospective study that hemilaminectomy was a safe approach and resulted in a total excision rate of 93% for 164 intradural extramedullary spinal tumors.

Predictors of incomplete resection in our study included preoperative functional impairment (mMCS >2), combined intra- and extramedullary tumor location, and tumors located in the cervical or thoracic spine. Tumors with both a relevant intramedullary and extramedullary tumor portion might also have a larger tumor volume.

#### 5.1.1 Postoperative bleeding

Postoperative hemorrhage is a rare but significant complication. In our study, bleeding occurred in 3.9% of patients, with no significant difference between the two surgical approaches. However, involvement of two or more spinal segments was a strong independent predictor of postoperative bleeding. This finding is in line with a previous investigation by Muncan et al. (2024) analyzing 187 patients with spinal schwannomas or meningiomas and also found no relevant difference regarding postoperative complications. Furthermore, Dobran et al. (2021) retrospectively analyzed 40 patients and also found no significant difference in complication rates between hemilaminectomy and laminectomy when treating spinal schwannomas, reinforcing that the tumor size rather than the approach itself may influence bleeding risk. Blood or fluid collection is also correlated with the invasiveness of the initial procedure and Lee et al. (2015) found a significantly reduced blood loss of 207 ml in their hemilaminectomy group compared with 426.6 ml in those being treated with laminectomy for spinal schwannomas. Furthermore, length of stay and operative time were significantly reduced in the hemilaminectomy group.

#### 5.1.2 Functional outcomes

The functional status and preoperative neurological burden were comparable at baseline among those being treated via a laminectomy or a hemilaminectomy. At the 12-month follow-up, functional status was comparable between these surgical approaches, with the majority of patients maintaining or improving their preoperative condition. The proportion of patients with a modified McCormick Scale score of I-III was 82.7% in the laminectomy group and 81.2% in the hemilaminectomy group, with no significant difference (p = 0.97). These results align with prior studies on intradural tumor resections, where functional outcomes were found to be independent of the surgical approach (Goodarzi et al., 2020). Muncan et al. (2024) found no significant difference in postoperative functional outcomes between laminectomy and hemilaminectomy for spinal schwannomas and meningiomas, with similar rates of neurological improvement based on the mMCS. The proportion of patients with unchanged neurological function was slightly higher in the laminectomy group (34.2%) than in the hemilaminectomy group (23.1%), though not statistically significant. Similarly, our study on spinal hemangioblastomas showed no significant difference in 12-month functional outcomes, reinforcing that both approaches effectively preserve neurological function across different tumor entities. Additionally, Goodarzi et al. (2020) concluded that hemilaminectomy offers advantages in preserving spinal stability while ensuring complete tumor resection. The shift from traditional open surgery to minimal invasive techniques has been widely adopted across various surgical fields, driven by technological advancements (Kong et al., 2022; Wang et al., 2015). In spinal surgery, minimally invasive approaches have been successfully implemented for conditions such as lumbar spinal stenosis, demonstrating outcomes comparable to standard laminectomy (Thomé et al., 2005; Nerland et al., 2015). Similarly, in spinal sHB surgery, hemilaminectomy seems to be a less invasive alternative to laminectomy, aiming to preserve spinal stability while ensuring complete tumor resection. The study by Krüger et al. (2019) has shown that minimally invasive resection via hemilaminectomy using tubular retractors is both feasible and effective, achieving high rates of complete resection with preserved neurological outcomes. However, as seen in our study, both approaches resulted in comparable resection rates and functional outcomes, reinforcing the growing evidence that minimally invasive techniques can achieve oncological efficacy without compromising neurological recovery.

### 5.2 Limitations

Despite this study is the largest multicentric investigation comparing laminectomy or hemilaminectomy for the resection of a homogeneous spinal intradural tumor entity, the present analysis has several limitations. First, this comparative study is of retrospective nature and not a controlled randomized trial. Second, primary objective of this international multicenter study was the investigation of progression-free survival and neurological outcome. Third, lack of long-term spinal stability and deformity data represents a relevant limitation, particularly for multilevel laminectomy. While preservation of posterior elements is a theoretical advantage of hemilaminectomy, the present study did not include systematic long-term radiographic assessment of spinal alignment or instability. Therefore, potential differences in postoperative kyphosis or instability cannot be assessed. Furthermore, the results from this retrospective investigation might be confounded by potential selection bias because treating surgeons might be prone to perform laminectomy in more extensive and multisegmental tumors. Notably, multilevel and thoracic tumors were more frequently treated by laminectomy, suggesting case-complexity–driven selection. Estimated intraoperative blood loss was not uniformly documented across all centers and could therefore not be analyzed; future prospective studies should include standardized recording of EBL and transfusion requirements. Because this prespecified secondary analysis was designed around approach-related perioperative endpoints and 12-month functional outcome, we did not perform a detailed analysis of long-term recurrence/local progression or de novo lesion development, which is particularly relevant in VHL patients with multifocal disease. These long-term disease-control endpoints have been addressed in our prior multicenter work and biomarker subgroup analysis (Wach et al., 2025a, 2025b). Preoperative spinal angiography and embolization were not captured in the standardized multicenter data entry template and could therefore not be analyzed; we plan to include these variables in a follow-up study to evaluate their impact on perioperative outcomes, including postoperative bleeding (Butenschoen et al., 2023)**.**

## 6 Conclusions

Both laminectomy and hemilaminectomy provide comparable rates of complete resection and long-term functional outcomes in the surgical treatment of spinal hemangioblastomas. Postoperative bleeding was significantly associated with multisegmental tumor involvement, while the choice of surgical approach did not influence complication rates.

## Informed consent

Informed consent was not required due to study design requiring no collection of data other than from the published literature.

## Code availability

Not applicable.

## Ethical approval

The study was conducted in accordance with ethical standards and received approval from the Leipzig University Ethics Committee (No.: 382/23-ek).

## Ethics approval/study registration

The study was conducted in accordance with ethical standards and received approval from the Leipzig University Ethics Committee (No.: 382/23-ek).

## Author contributions

Data analysis: J.W., E.G., A.E.B.; Manuscript drafting: J.W., E.G., A.E.B.; Supervision: E.G.; Review–Editing: All authors; Study design: J.W., E.G., A.E.B., T.T.; Data acquisition: All authors contributed to acquisition and collection of data; Statistical analysis: J.W., E.G., A.E.B.

## Funding

Not applicable.

## Declaration of competing interest

The authors declare that they have no known competing financial interests or personal relationships that could have appeared to influence the work reported in this paper.

## Data Availability

The datasets generated and analyzed in the current study are available form the corresponding author on reasonable request.
