# Supplementary material for: Comparing laminectomy and unilateral hemilaminectomy in spinal hemangioblastoma resection: A multicenter study
Source: Brain Spine. 2026 Mar 5;6:106004. doi: 10.1016/j.bas.2026.106004 (PMC12993167; doi:10.1016/j.bas.2026.106004)
Supplement: Multimedia component 1 [file mmc1.pdf]

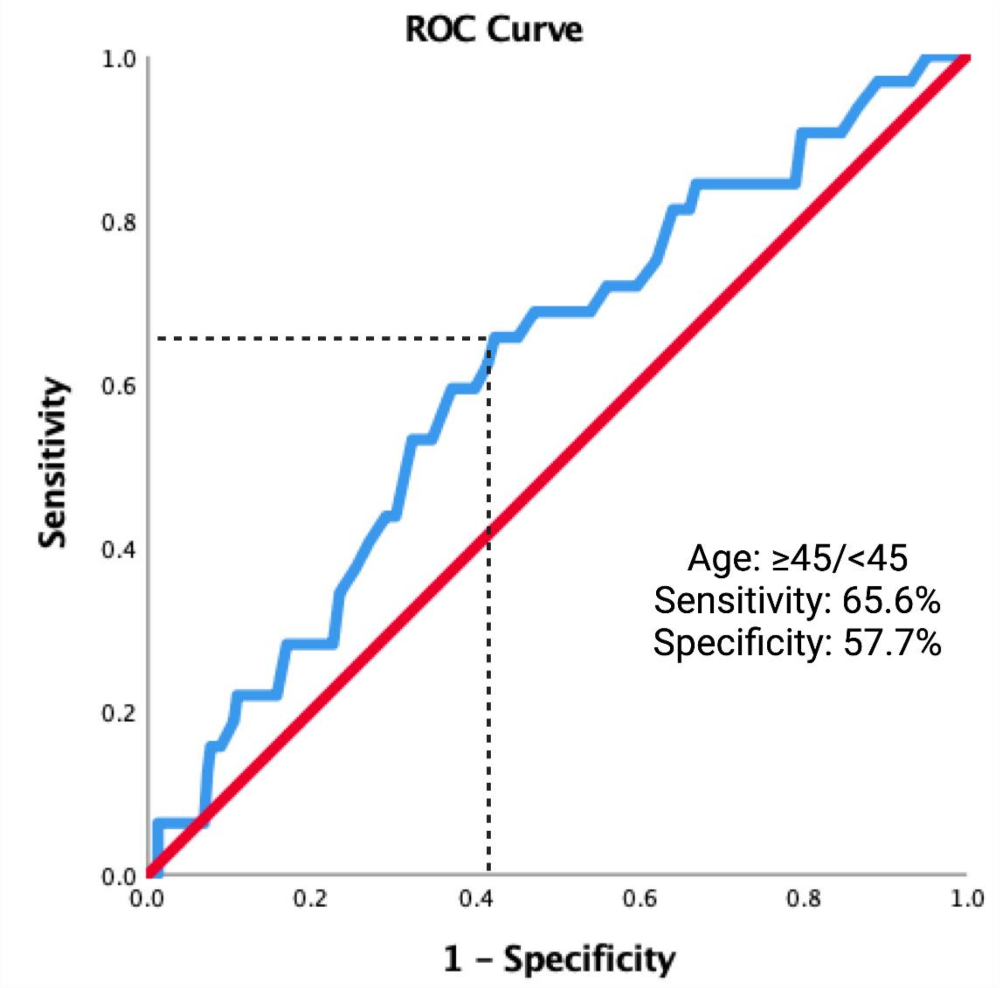

Supplementary figure 1. ROC curve analysis illustrating diagnostic accuracy of age in determining extent of resection (incomplete or complete resection).
