# Supplementary material for: Comparing laminectomy and unilateral hemilaminectomy in spinal hemangioblastoma resection: A multicenter study
Source: Brain Spine. 2026 Mar 5;6:106004. doi: 10.1016/j.bas.2026.106004 (PMC12993167; doi:10.1016/j.bas.2026.106004)
Supplement: Multimedia component 2 [file mmc2.docx]

**Supplementary table 1: STROBE Checklist – (Manuscript: Laminectomy vs. Hemilaminectomy for sHB)**

| **Item No.** | **Recommendation** | **Addressed in Manuscript** | **Page(s)** |
| --- | --- | --- | --- |
| **Title and abstract** |  |  |  |
| 1(a) | Indicate the study’s design in the title/abstract | Abstract: “Retrospective international multicenter study” | p. 1 |
| 1(b) | Informative, balanced summary | Abstract describes background, objectives, methods, results, and conclusion | p. 1 |
| **Introduction** |  |  |  |
| 2 | Background/rationale | Explains rarity of sHB, surgical challenges, gap in literature | p. 2 |
| 3 | Objectives | Compare resection, outcomes, bleeding; prespecified endpoints | p. 2 |
| **Methods** |  |  |  |
| 4 | Study design | “Retrospective international multicenter study” early in Methods | p. 3 |
| 5 | Setting | 13 centers (US, Europe), recruitment from pooled databases, 12-month follow-up | p. 3 |
| 6(a) | Participants: eligibility & follow-up | Inclusion: primary sHB, laminectomy or hemilaminectomy; exclusion: recurrence, laminoplasty, fixation | p. 3–4 |
| 6(b) | Matching criteria | Not applicable (no matching) | — |
| 7 | Variables | Defined outcomes: extent of resection, mMCS, postoperative bleeding; predictors | p. 4–5 |
| 8 | Data sources/measurement | Institutional databases, MRI, functional status by neurosurgeons/radiologists | p. 4–5 |
| 9 | Bias | Notes possible selection bias; retrospective limitations | p. 17 |
| 10 | Study size | n=280 after screening; no formal sample size calculation | p. 3, 6 |
| 11 | Quantitative variables | Age dichotomized at 45 y via ROC; other categorical groupings | p. 6–7 |
| 12(a) | Statistical methods | Chi-squared/Fisher/t-test, ROC, logistic regression | p. 5–6 |
| 12(b) | Subgroups/interactions | Subgroup: hemilaminectomy vs. laminoplasty | p. 15 |
| 12(c) | Missing data | Preoperative mMCS available for 276 pts; no imputation | p. 7, Table 1 |
| 12(d) | Loss to follow-up | 12-month follow-up reported; no major loss noted | p. 13 |
| 12(e) | Sensitivity analyses | Not reported | — |
| **Results** |  |  |  |
| 13(a) | Participants: numbers | Flowchart (Fig. 1); n=357 screened, n=280 included | p. 6 |
| 13(b) | Reasons for non-participation | Prior surgery (n=38) or additional procedures (n=39) | p. 6 |
| 13(c) | Flow diagram | Fig. 1 | p. 6 |
| 14(a) | Descriptive data | Table 1: demographics, tumor location, levels | p. 7 |
| 14(b) | Missing data | Reported for mMCS | p. 7 |
| 14(c) | Follow-up time | 12 months | p. 5, 13 |
| 15 | Outcome data | Resection rates, bleeding events, functional status at follow-up | p. 8–14 |
| 16(a) | Main results | Adjusted ORs, 95% CI; confounders stated | p. 8–10 |
| 16(b) | Category boundaries | e.g., age <45 vs. ≥45 y | p. 8 |
| 16(c) | Absolute risks | Not converted to absolute risk | — |
| 17 | Other analyses | Hemilaminectomy vs. laminoplasty subgroup | p. 15 |
| **Discussion** |  |  |  |
| 18 | Key results | Restates primary findings | p. 16 |
| 19 | Limitations | Retrospective, selection bias, lack of long-term stability data | p. 17 |
| 20 | Interpretation | Contextualized with literature; cautious interpretation | p. 16–18 |
| 21 | Generalisability | Notes alignment with other tumor types and approaches | p. 18 |
| **Other information** |  |  |  |
| 22 | Funding | No funding; no funder role | p. 20 |
