# Supplementary material for: Comparing laminectomy and unilateral hemilaminectomy in spinal hemangioblastoma resection: A multicenter study
Source: Brain Spine. 2026 Mar 5;6:106004. doi: 10.1016/j.bas.2026.106004 (PMC12993167; doi:10.1016/j.bas.2026.106004)
Supplement: Multimedia component 3 [file mmc3.docx]

| **Supplementary Table 2.** Subgroup comparative analysis between Hemilaminectomy and Laminoplasty | | | |
| --- | --- | --- | --- |
| Variable | Hemilaminectomy (n = 155) | Laminoplasty (n = 34) | *p*-value |
| Age, mean years (SD) | 42.5 +/- 15.7 | 44.2 +/- 18.3 | 0.59 |
| Female, n patients (%) | 79 (51.0%) | 17 (50.0%) | 0.92 |
| Modified McCormick, preoperative, n patients (%), *  I-II  III-V | 111 (73.0%)  41 (27.0%) | 27 (79.4%)  7 (20.6%) | 0.52 |
| Location  Cervical  Cervicothoracic  Thoracic  Thoracolumbar  Lumbar  Lumbosacral | 74 (47.4%)  12 (7.7%)  36 (23.2%)  21 (13.5%)  8 (5.2%)  4 (2.6%) | 21 (61.8%)  0 (0.0%)  10 (29.4%)  1 (2.9%)  2 (5.9%)  4 (2.6%) | 0.19  0.13  0.51  0.14  0.99  0.99 |
| Levels operated, n (%)  1  >1 | 104 (67.1%)  51 (32.9%) | 11 (32.4%)  23 (67.6%) | 0.0001 |
| Location  Intramedullary  Extramedullary  Combined | 113 (72.9%)  19 (12.3%)  23 (14.8%) | 15 (44.1%)  13 (38.2%)  6 (17.6%) | 0.002  0.0008  0.98 |
| Extent of resection  Complete  Incomplete | 140 (90.3%)  15 (9.7%) | 29 (85.3%)  5 (14.7%) | 0.37 |
| Outcome group at 12-months (%), *  Good  Poor | 88 (87.1%)  13 (12.%) | 23 (79.3%)  6 (20.7% | 0.37 |
| Postoperative bleeding | 5 (3.2%) | 0 (0.0%) | 0.59 |
| * available in 186 patients | | | |
